# PD92 Health Technology Assessment Of Genetic And Genomic Applications: A Proposal For A Standardized Approach

**DOI:** 10.1017/S0266462324003398

**Published:** 2025-01-07

**Authors:** Antonio Sciurti, Giuseppe Migliara, Valentina Baccolini, Erika Pitini, Anna Ewa Kaminska, Valentina Soccodato, Ilaria Mussetto, Carolina Marzuillo, Paolo Villari

## Abstract

**Introduction:**

Frameworks based on the ACCE model, which have a limited health technology assessment (HTA) approach, have long been used in the assessment of genetic and genomic applications (GGAs). While ACCE frameworks are mainly focused on technical aspects, HTA assessments include economic and organizational aspects. The aim of this study was to develop a framework able to address a comprehensive assessment of GGAs.

**Methods:**

We analyzed the most currently used HTA model in Europe, the EUnetHTA HTA Core Model®, to investigate its suitability as an HTA framework for GGAs and to identify any possible shortcomings in its evaluation. The Sapienza evaluation framework, which is based on a previous systematic review of assessment frameworks for GGAs, was used as the comparison. The HTA Core Model applications were then used as a basis for an assessment framework for GGAs, classified according to “GGA function/use setting” pairs.

**Results:**

The domains included in the Sapienza framework fully corresponded with the HTA Core Model. In addition, the “Screening Technology” application of the HTA Core Model was found to be suitable for diagnostic, pre-symptomatic, predictive, and carrier GGAs when used in screening settings. The “Diagnostic Technologies” application was deemed appropriate for the pairs “Diagnostic/Diagnosis”, “Pre-symptomatic/Diagnosis”, “Prognostic/Staging”, and “Pharmacogenetic/Staging”, although it may require additional considerations such as the need for screening in the case of germline mutations, the use of appropriate accuracy measures for prognostic GGAs, and the evaluation of the clinical utility of pharmacogenetic or prognostic GGAs.

**Conclusions:**

The proposed approach is a flexible tool that allows assessment of GGAs using a shared and validated methodology that considers the technical aspect, clinical utility, economic value, and delivery models. We found that the HTA Core Model fits the needs of GGA assessment, although some GGA peculiarities should be further explored in the assessment process.

